# Drug-likeness of Phytic Acid and Its Analogues

**DOI:** 10.2174/1874285801509010141

**Published:** 2015-11-03

**Authors:** Amitha Joy, S. Balaji

**Affiliations:** 1 Department of Biotechnology, Sahrdaya College of Engineering and Technology, Kodakara-680684, Thrissur, India;; 2 R&D Centre, Bharathiar University, Coimbatore, Tamilnadu, 641046, India;; 3 Department of Biotechnology, Manipal Institute of Technology, Manipal 576104, Karnataka, India

**Keywords:** Analogues, binding energy, bioactivity, docking, druglikeness, phytic acid

## Abstract

Inositol hexakisphosphate is known to be the phosphorous reserve in plants particularly in the seeds. Though it has been known for its antinutrient properties for many years, recent research shed light to reveal it as a novel anticancer agent. Hence the present study investigates the drug-likeness of phytic acid and its analogues through bioinformatics methods. Two potential cancer drug targets such as mitogen activated kinase and inositol 1,4,5-triphosphate receptor are included in the study. Out of 50 selected analogues of phytic acid, 42 structures interact well with the chosen drug targets. The best interacting structures are 1-diphosinositol pentakisphosphate and 2,3,4,5,6-pentaphosphonooxycyclohexyl dihydrogen phosphate. For both of these structures, the negative binding energy obtained was -49.5 KJ/mol; this affirms the stability of the complex. ADME properties are also predicted to assess the drug-like properties of the compounds. The structure activity relationship model is generated for 12 compounds with experimental IC50 values.

## 1 INTRODUCTION

Inositol hexakisphosphate (InsP6), also known as phytic acid is the foremost inositol phosphate discovered [1]. It is the most abundant organic phosphate in the world [2]. In plants, InsP6 acts as a phosphate reserve, especially in the seeds [3]. In nature phytic acid (C_6_H_18_O_24_P_6_) can exist as free acid, phytate or phytin, depending on the physiological pH and metal salts [4]. The percentage of phytate in many of the plants has been quantified [5] and their antinutrient properties are well studied [6]. Recent research investigations have demonstrated its activity against cancer cell lines such as human colon cancer cell line (HT-29), erythroleukemic cell line (K-562), human liver cancer cell line (HepG2), human prostate carcinoma cell line (DU 145) and rhabdomyosarcoma cell line (RD) [7, 8]. The pessimistic view of phytic acid in human diet is optimistically assigned for human health due to its anti-cancer activities in colon, prostate, metastatic and breast cancers. It is also used to chelate and clear renal stones [9]. Besides *in vitro* studies, the *in vivo* studies using animal models support anticancer effects of phytic acid and hence phytic acid is referred to as ‘natural cancer fighter’ [10]. However the drug-likeness of phytic acid is not well documented in literature, some more research investigations and clinical trials need to be done to prove its drug-likeness. Computational methods play a vital role in the screening of lead molecules, designing novel drug molecules, identifying potent protein targets and also to understand the mechanism of drug action in greater detail. In particular docking and QSAR studies are effective for understanding the structural features of lead molecules. *In silico* approaches can assist**in this regard to study the interactions of phytic acid with various drug targets. At present there is no published research work on the structure-activity relationship (SAR) of phytic acid and its analogues. Elucidating SAR can help in screening out novel inhibitors that can bind with the druggable targets. Hence the present study makes an attempt to bring forth the drug-like properties of phytic acid and its analogues through computational approach.

## 2 METHODOLOGY

### 2.1 Ligand Preparation

The 2D structures of phytic acid and its analogues were retrieved from the Pubchem repository of NCBI [11]. Ligprep module [12] was used to prepare ligands. It employed series of steps that performed 3D conversions, corrections and optimization of the following structures. The Pubchem accession numbers are given below 

CID477, CID890, CID107758, CID125004, CID127297, CID178749, CID439456, CID443266, CID2524165, CID4200706, CID4487899, CID10251645, CID10747577, CID14375662, CID16752671, CID16752673, CID18365880, CID21099914, CID44274820, CID44332437, CID45479488, CID46173206, CID46173281, CID46173316, CID46173429, CID46173525, CID46905360, CID52949527, CID53379838, CID53380009, CID53380097, CID53380098, CID53380099, CID53380100, CID53380197, CID53380199, CID53380200, CID53380300, CID53380301, CID53380721, CID53380834, CID53380835, CID53380836, CID53380837, CID53462026, CID53477671, CID54002314, CID54207372, CID57503246 and CID57773931.

### 2.2 Identification and Preparation of Protein Targets

Identification of a protein target is a crucial step in interaction studies. In the present work one such target was selected using PASS online server [13]. The server predicts several pharmacological effects and biochemical mecha-nisms on the basis of structural formula of a substance. The prediction results are analyzed based on the activity values. The compound is very likely to reveal the activity in experimentation if the activity value > 0.7. If the compound is between 0.5 and 0.7 it is expected to exhibit the activity in experiment but the likelihood is less. The substance is improbable to display the activity in experiment if the activity value is < 0.5. Based on the PASS results Inositol 1,4,5-triphosphate receptor was selected as a protein target. Literature survey suggested one more potent drug target for colon cancer mitogen activated protein kinase [14, 15]. Their corresponding PDB ids 1N4K and 1PMQ were chosen for the study. The crystal structures of the two receptors were prepared using Proteinprep wizard of Schrödinger Release 2013-1 [16]. The PDB file included a co-crystallized ligand and does not include explicit hydrogens. The structures were prepared by adding hydrogens appropriately to get refined structures. 

### 2.3 Grid Generation and Docking

Grid generation was done using the Glide module of Schrödinger Release 2013-1 [17]. The ligand poses produced are subjected to move across a chain of ranked screens that estimate the ligand’s interaction with the receptor. In grid generation, pose refers to a whole measurement of the ligand location and orientation relative to the receptor core conformation as well as rotamer set conformations. Receptor atom van der Waals radii can be scaled and either partial charges from the force field or from the input structure can be chosen. The maximum size of the enclosing grid box was set as 50Å. The process of docking a ligand into a binding site tries to generate low energy conformations. The molecules are brought together by assigning planes that contain the sites of interaction and then moving the planes while calculating binding energies of interaction [18]. Here docking was done using Glide module. Glide uses a series of hierarchical filters to search for possible locations of the ligand in the active-site region of the receptor. The shape and properties of the receptor are represented on a grid by different sets of fields that provide progressively more accurate scoring of the ligand poses [19].

### 2.4 Prime MM-GBSA

The ligand binding energies were calculated using molecular mechanics with generalized Born and surface area solvation (Prime MM-GBSA) [20]. Combinations of the docking method with other methods, such as MD simulation and free energy binding calculation enables rational drug design [21]. Solvation plays an important role in molecular recognition that was considered in the MM-GBSA scoring [22]. Prime MM-GBSA predicts various energies of the complex such as coulomb energy, covalent binding energy, van der Waals energy, surface area, solvation energy, prime 

energy, mmgbsa energy, mmgbsa energy of the free ligand, mmgbsa energy of the uncomplexed receptor, mmgbsa energy of the ligand in the complex, mmgbsa ligand strain energy and mmgbsa free energy of binding excluding ligand strain.

### 2.5 Computational Prediction of ADME Properties

Absorption, distribution, metabolism, and excretion (ADME) properties of phytic acid and its derivatives were predicted by QuikProp [23]. QuikProp also predicts physically significant descriptors and pharmaceutically relevant properties for organic structures, either individually or in batches. The QuikProp predictions are for orally delivered drugs and for non-active transport. It also checks the Lipinski’s rule of five [24]. 

### 2.6 QSAR Studies

Quantitative structure activity relationship of phytic acid and its derivatives were predicted by using GUSAR software. It helps to create QSAR models on the basis of the appropriate training sets [25]. It used a self-consistent regression (a regularized least-squares method) for building QSAR models [26]. Out of the 50 structures, 12 were selected for the studies for which the experimental IC_50_ values were available in Pubchem database. Based on the training set, IC_50 _values were predicted using quantitative neighbourhood of atoms (QNA model) and multilevel neighbourhood of atoms (MNA model) [27]. On the basis of QNA descriptors the involvement of every atom corresponding to its experimental activity is displayed. The predicted value correlates with the activity of the whole molecule is indicated by green, blue implies that the predicted value is below the activity of the whole molecule and red indicates that the predicted value is greater than that of the whole molecule. 

## 3 RESULTS AND DISCUSSION

The structure of phytic acid was very likely to be active against 46 targets, Table **1**. It is instinctive as well as evident that phytic acid is an inositol 1,4,5-triphosphate receptor 1 antagonist. This is in concord with Sakakura *et al*., they suggested inositol 1,4,5-triphosphate receptor as a molecular target for gastric cancers [28]. The crystal structure of inositol 1,4,5-triphosphate receptor in complex with IP_3 _is available in PDB (1N4K). Inositol 1,4,5-triphosphate receptor is involved in calcium signalling process. Calcium signaling is a vibrant signalling pathway which regulates numerous cellular processes, including fertilization, cell growth, transformation, secretion, smooth muscle contraction, sensory perception and neuronal signaling [29]. Binding in the IP3 receptor therefore affects the calcium signalling pathway. MAPKs (Mitogen activated kinases) are involved in directing cellular responses to a diverse array of stimuli. They regulate cell functions including proliferation, gene expression, differentiation, mitosis, cell survival, and apoptosis [30]. There are several cancer drugs which target on MAPKs [31, 32]. 

### 3.1 Docking Results

Docking was carried out using phytic acid and its analogues using the targets, 1N4K and 1PMQ. The results show that all the selected compounds effectively bind with the selected targets. 1-diphosphoinositol pentakisphosphate (46173525) gave the best docking score of -14.29 for 1PMQ and 2,3,4,5,6-pentaphosphonooxycyclohexyl dihydrogen phosphate (178749) gave the best docking score of -14.02 for 1N4K. Table **2 **and Table **3** show the top ten docked poses. Out of fifty compounds, for 1PMQ forty two gave docking scores less than -10 and for 1N4K forty scored less than -10 which shows both the protein targets have good binding affinity towards the selected compounds. All the ligands which scored less than -10 have hydrogen bond donor atom count ranging from 8 to 14, hydrogen bond acceptor atom count ranging from 12 to 30 and heavy atom count ranging from 24 to 44 according to Pubchem repository [11]. These features would have influenced the binding affinity of the ligands. All the poor binders have defined and undefined steriocentred atom count ranging from 4 to 6. This sterioisomerism also would have been responsible for their poor binding affinity. Stereochemistry in drug action is of great importance in medical practice [33]. Chiral drugs have two structurally similar forms that can act in a different way in biological systems owing to their different shapes in 3-dimensional space. In a chiral environment, one enantiomer may exhibit different chemical and pharmacological behavior than the other enantiomer and the difference in 3-dimensional structure of the inactive enantiomer prevents them from having a biological effect at this binding site [34]. This explains the impact of stereoisomerism on binding affinity. 

The amino acids involved in the binding site of 1N4K include tyrosine 567, arginine 511, arginine 265, lysine 508, arginine 269, glycine 268, lysine 569, arginine 568 and threonine 267. The amino acids involved in the binding site of 1PMQ include lysine 93, tyrosine 223, lysine 191, valine 225, glutamine 255, alanine 74, aspartate 189, valine 224, arginine 107, threonine 226, serine 193, serine 72, glycine 71, valine 78 and aspargine 194. The amino acids in the binding pocket of both the proteins have some residues in common, like tyrosine, arginine, threonine, lysine and glycine. Glide scoring function is given by the equation XP GlideScore = Ecoul + EvdW + Ebind + Epenalty [35, 36] where XP refers to the extra precision mode, its scoring function is given by the sum of the coulombic interaction energy, van der Waals energy, binding energy and penalty which includes factors that hinders binding. A pictorical representation of the interaction between phytic acid and 1PMQ receptor Fig. (**1**).

### 3.2 MMGBSA Prediction

The result of Prime MMGBSA distinguishes strong and weak binders (Table **4**). The free energy of binding was least for (1r,2R,3S,4ss,5R,6S)-2,3,4,5,6-pentakis (phosphonooxy) cyclohexyl tetrahydrogen triphosphate (53477671). This ligand has 8 phosphate groups available for binding when compared with others. It also pocess maximum numbers of hydrogen bond donor atom count, acceptor atom count, heavy atom count and complexity values of 14, 30, 44 and 1340 respectively [11] when compared with the other ligands. The coulombic energy of the compound (-38.8kcalmol^-1^), van-der-waal’s energy (-40.1 kcalmol^-1^), co-valent energy (-21.6 kcalmol^-1^), coulombic binding energy (-11092.4 kcalmol^-1^) and solvation binding energy (-2168.6 kcalmol^-1^) were also significantly low. The presence of aromatic group and hydroxyl group bearing tyrosine, phosphate anion binder arginine and highly reactive amino group bearing lysine in the binding pocket of both the targets also account for the binding efficacy and binding energy minima [37]. The compound 2,3,4,5,6-pentaphospho-nooxycyclohexyl dihydrogen phosphate (CID178749) ranked in the top docked poses in both the protein targets have a satisfactory binding energy minima of -12.8 kcalmol^-1^. This compound is otherwise known as technetium-99m. Technetium-99m is a radioisotope most widely used in medicine employed in over half of all nuclear medicine procedures [38]. 

Thirty one of the compounds gave minimum energy values. The presence of hydroxyl and phosphate functional groups in these compounds enhanced their binding affinity. The negative binding free energies showed favourable ligand protein complexes. 

### 3.3 ADME Screening

According to the Qikprop analysis 44 physically significant descriptors and pharmaceutically relevant properties of the ligands like molecular weight, total solvent accessible surface area (SASA), hydrophobic and hydrophilic component and volume were obtained. The Table **5** shows some of the predicted properties. Natural products are frequently cited as exclusion to Lipinski's rules because environment has learned to sustain low hydrophobicity and intermolecular H-bond donating ability when it needs to make biologically active compounds with high molecular weight and large numbers of rotatable bonds [39]. 

### 3.4 Quantitative Structure Activity Relationship Study Using GUSAR

QSARs are mathematical models that attempt to relate the structure-derived features of a compound to its biological or physicochemical activity. QSAR works on the assumption that structurally similar compounds have similar activities [40]. In the present work, in the consensus QSAR model given by GUSAR. In the present study majority of the selected compounds correlate with experimental and predicted activity. Three models were generated by GUSAR- QNA model, MNA and Combinatorial model as given in Table **6**. For QNA model and Combinatorial model the predicted IC_50_ values were very less deviated from the experimental IC_50. _QNA model gave r^2^ (goodness of fit) of 0.943 and Combinatorial model gave the best result with r^2^ value 0.960. For MNA model the r^2^ value is 0.660.

The statistical characteristics of the model with high value of r^2^, q^2^, F and low value of SD and the equation indicates that the model is statistically significant, could be used for analogue screening. The regression equations for the MNA model, QNA model and combinatorial model are 1.33x-7.298, 0.899x + 0.419 and 0.836x – 0.303 respectively indicates a linear relationship between experimental IC_50_ value and predicted IC_50_ value. Therefore phytic acid and the selected analogues for GUSAR studies have similar activities. 

## CONCLUSION

The present work identified mitogen activated kinase and inositol 1,4,5-triphosphate receptor as the protein targets for phytic acid. The interaction (docking scores) are -14.29 and -14.02, respectively. The protein ligand complexes had a minimum binding energy of -49.5kJ/mol. The parent compound, phytic acid and its similar compounds resemble similar mode of binding due to chemical similarity. The amino acid pattern involved in the binding interaction is almost same for all the compounds. The analyzed compounds are predicted to be drug-like. The structure activity relationship model correlated the atom level contributions to the activity. This would be made beneficial for designing novel drug compounds. Furthermore animal trials and clinical studies and can reveal more insights into the drug-likeness of phytic acid. 

## Figures and Tables

**Fig. (1):**
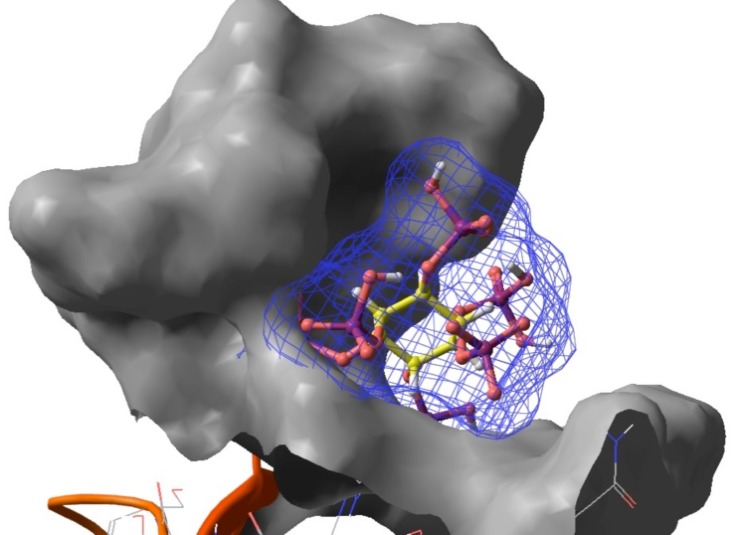
Interaction of phytic acid (sticks with a mesh surface) with the binding site of the receptor 1PMQ (solid surface), rendered using Maestro (a product of Schrödinger, Inc. [16]).

**Table 1:** Predicted biological activity spectrum of phytic acid.

Sl No.	Pa	Pi	Activity
1	0,986	0,000	Inositol 1,4,5-triphosphate receptor 1 antagonist
2	0,981	0,002	Tubulin antagonist
3	0,959	0,003	Angiogenesis inhibitor
4	0,954	0,000	Sphingosine 1-phosphate receptor 5 antagonist
5	0,909	0,004	Sugar-phosphatase inhibitor
6	0,904	0,003	Bisphosphoglycerate phosphatase inhibitor
7	0,897	0,009	Aspulvinonedimethylallyltransferase inhibitor
8	0,882	0,003	Ribulose-phosphate 3-epimerase inhibitor
9	0,859	0,007	Mannotetraose 2-alpha-N-acetylglucosaminyltransferase inhibitor
10	0,818	0,010	Glucose oxidase inhibitor

Pa- probability to be active; Pi- probability to be inactive.

**Table 2:** Docked ligands in the order of XP score (1N4K).

Pubchemid	SP score	XP score	Emodel	Glide energy	Evdw	Ecoul
178749	-14.00	-14.02	-55.19	-101.78	-27.11	-103.71
4200706	-12.47	-12.50	-38.07	-83.45	-4.36	-83.01
16752671	-11.79	-12.46	10000.00	-47.57	-25.11	-75.10
46173525	-11.68	-12.36	10000.00	-43.96	-4.36	-63.20
46173281	-11.68	-12.36	10000.00	-43.96	-32.28	-63.20
107758	-11.51	-12.22	-116.22	-80.52	-10.61	-73.76
21099914	-12.19	-12.21	10000.00	-49.63	-25.73	-81.18
890	-11.93	-11.96	10000.00	-63.94	-14.24	-57.45
57773931	-11.14	-11.83	-43.24	-85.29	-6.11	-79.26
53380198	-11.77	-11.80	10000.00	-29.84	-18.00	-75.21

SP- Single precision, XP- extra precision, Emodel- weighting of forcefield components, Evdw- vander waal’s energy, Ecoul- coulombic energy.

**Table 3:** Docked ligands in the order of XP score (1PMQ).

Pubchemid	SPscore	XP score	Emodel	Glide energy	Evdw	Ecoul
46173525	-5.27	-14.29	-79.45	-58.95	-19.44	-39.51
53380834	-6.12	-13.06	-0.08	-98.38	-28.90	-37.13
4487899	-6.41	-12.98	-95.79	-81.79	-20.02	-61.77
53379838	-7.11	-12.77	-73.91	-84.42	-27.11	-57.31
178749	-5.46	-12.48	-46.13	-51.94	-8.89	-43.05
477	-5.8	-12.4	-82.29	-64.78	-25.11	-39.67
125004	-5.47	-12.32	-68.41	-68.04	-4.36	-63.68
45479488	-5.64	-11.99	-82.29	-64.78	-25.11	-39.67
53477671	-5.86	-11.95	-89.23	-72.58	-10.09	-62.50
10747577	-5.73	-11.77	-68.41	-68.04	-4.36	-63.68

**Table 4:** MM-GBSA prediction.

Pubchemid	Binding Free Energy (kcalmol-1)	Coulombic Energy of Complex (kcalmol-1)	Van der Waals Energy of the Complex (kcalmol-1)	Covalent Energy (kcalmol-1)	Coulombic Binding Energy (kcalmol-1)	Solvation Binding Energy (kcalmol-1)
53477671	-49.5	-38.8	-40.1	-21.6	-11092.4	-2168.6
53380834	-44.6	-51.4	-36.2	7.0	-10964.1	-2146.9
53380009	-40.3	-14.1	-49.0	-10.5	-10982.1	-2169.7
53380100	-37.0	-16.4	-49.9	-13.2	-10985	-2161.7
53380199	-34.5	-15.0	-35.7	-12.4	-10958.1	-2195.5
53380835	-33.4	-2.4	-42.7	4.1	-10912.6	-2184.5
46173525	-30.1	-16.2	-37.7	-12.6	-11022.8	-2193.3
25245165	-30.0	35.6	-24.4	-22.6	-10999.6	-2257.0
53380837	-28.7	-42.2	-27.7	2.9	-10963.3	-2141.5
53380300	-22.2	-26.3	-31.0	-10.2	-10974.4	-2172.3
53380836	-21.6	-33.6	-36.9	3.6	-10952.3	-2152.5
53380098	-21.2	-21.5	-41.1	-5.9	-10984.4	-2152.3
53380198	-19.0	-46.5	-46.7	-5.8	-10923.2	-2127.8
53380097	-18.3	-3.3	-51.8	-12.0	-10970.7	-2162.9
4487899	-17.1	-17.3	-32.9	-16.7	-11037.6	-2180.2
53379838	-16.3	-27.3	-35.3	-12.9	-11064	-2180.0
53462026	-14.6	4.5	-28.2	-15.6	-11034.4	-2202.5
53380197	-14.5	42.8	-41.9	-4.6	-10915.6	-2213.4
45479488	-14.5	-14.2	-30.7	-10.4	-11044.8	-2193.9
127297	-14.1	37.4	-40.0	-10.3	-11017.1	-2216.8
185839	-12.8	7.8	-36.5	-21.5	-10976.9	-2207.2
178749	-12.8	7.8	-36.5	-21.5	-10976.9	-2207.2
46173206	-12.0	-22.4	-21.4	-21.2	-11037.2	-2184.6
46173429	-10.0	-14.2	-19.6	-16.7	-11070.1	-2172.9
53380200	-9.1	14.4	-47.7	-7.3	-10940.1	-2181.8
53380099	-8.8	8.0	-37.7	-1.4	-10967.2	-2171.5
46173281	-8.6	-17.4	-22.9	-26.4	-11030.5	-2183.0
52949527	-6.8	4.1	-25.5	-19.3	-11021.6	-2196.5
23675791	-5.8	9.7	-35.5	-21.7	-10969.9	-2206.7
10747577	-5.7	-21.4	-17.0	-13.5	-11041.8	-2187.5
16752673	-2.5	-5.5	-32.1	-14.4	-11018.8	-2177.1

**Table 5:** ADME properties prediction.

Pubchem id	Molwt	SASA	FOSA	FISA	QPlogBB	Volume	DonorHB $	AccptHB $	QPlogPo/w
890	660.0	659.2	22.1	616.2	-6.3	1279.9	12	30	-1.6
46173525	740.0	709.2	14.7	675.3	-6.3	1401.3	10	32	-1.7
107758	500.1	598.6	38.8	545.5	-7.2	1072.8	10	23.4	-2.2
4487899	740.0	720.8	24.4	673.3	-5.8	1407.8	10	32	-1.7
45479488	820.0	750.4	21.9	701.9	-7.2	1506.0	8	34	-1.7
16752671	740.0	740.5	5.6	712.5	-6.3	1436.9	10	32	-2.0
16752673	740.0	695.8	16.7	659.2	-6.1	1383.7	10	32	-1.6
46173525	740.0	709.2	14.7	675.3	-6.2	1401.3	10	32	-1.7
4200706	660.0	691.7	19.4	649.7	-6.5	1301.7	12	30	-2.0
178749	660.0	650.2	26.8	605.0	-6.4	1275.9	12	30	-1.4
53462026	820.0	738.7	16.0	701.2	-6.3	1504.0	8	34	-1.6
10747577	740.0	728.0	15.7	693.5	-6.1	1425.1	10	32	-1.8
53477671	820.0	771.7	38.7	720.7	-6.2	1532.5	10	36	-2.4
53380098	740.0	729.8	20.0	692.3	-6.5	1420.4	7	30.7	-2.1
53380099	740.0	757.7	20.9	717.7	-5.6	1469.2	7	30.7	-2.1
53380100	740.0	723.8	23.0	673.8	-7.3	1413.8	7	30.7	-1.9
53380197	740.0	703.3	23.9	658.4	-7.7	1403.5	7	30.7	-1.8
53380198	660.0	668.0	30.2	615.3	-7.9	1279.6	6	27.4	-2.1
53380199	740.0	781.5	33.6	729.0	-6.9	1483.1	7	30.7	-2.3
53380200	740.0	687.7	20.7	648.0	-7.2	1371.3	7	30.7	-1.8
53380300	740.0	689.4	38.1	630.7	-7.1	1368.7	7	30.7	-1.7
53380301	820.0	702.9	12.5	675.2	-6.1	1467.4	8	34	-1.4
53380097	740.0	745.2	42.7	680.3	-7.5	1437.8	7	30.7	-2.0
53380009	740.0	707.6	28.8	661.5	-7.6	1418.9	7	30.7	-1.7
25245165	820.0	772.7	6.6	749.1	-8.1	1535.9	8	34	-2.0
127297	820.0	718.5	15.8	676.6	-7.6	1470.5	8	34	-1.5
46173281	740.0	657.4	32.4	605.0	-8.0	1339.4	10	32	-1.1
46173429	820.0	750.1	26.4	701.4	-7.3	1495.3	8	34	-1.8
46173316	740.0	717.9	22.1	669.6	-6.9	1430.3	10	32	-1.5
46173206	740.0	692.4	15.3	658.4	-6.5	1387.0	10	32	-1.5
53379838	820.0	751.0	11.9	716.9	-8.5	1521.3	8	34	-1.7
53380837	740.0	748.8	25.7	696.5	-6.8	1457.9	4	29.4	-2.3
53380836	740.0	729.2	28.2	678.2	-6.6	1429.3	4	29.4	-2.3
53380835	740.0	706.2	33.7	652.2	-6.9	1387.4	4	29.4	-2.2
53380834	740.0	710.7	23.2	664.7	-7.6	1412.3	4	29.4	-2.2
53380721	740.0	749.4	28.1	692.3	-6.9	1428.6	7	30.7	-2.1
125004	740.0	687.7	20.7	648.0	-8.4	1371.3	7	30.7	-1.6
10251645	740.0	689.4	38.1	630.7	-7.1	1368.7	7	30.7	-1.6
14375662	820.0	702.9	12.5	675.2	-6.1	1467.4	8	34	-1.6
44274820	740.0	745.2	42.7	680.3	-7.8	1437.8	7	30.7	-2.0
44332437	740.0	707.6	28.8	661.5	-7.1	1418.9	7	30.7	-1.3
443266	820.0	772.7	6.6	749.1	-6.8	1535.9	8	34	-1.7
46905360	820.0	718.5	15.8	676.6	-7.8	1470.5	8	34	-1.6
477	740.0	657.4	32.4	605.0	-7.7	1339.4	10	32	-1.4
439456	820.0	750.1	26.4	701.4	-7.4	1495.3	8	34	-1.5

SASA- Total solvent accessible surface area (SASA) in square angstroms using a probe with a 1.4 Å radius. FOSA- Hydrophobic component of the SASA (saturated carbon and attached hydrogen). FISA- Hydrophilic component of the SASA (SASA on N, O, and H on heteroatoms). QPlogPo/w- Predicted octanol/water partition coefficient. QPlogBB -Predicted brain/blood partition coefficient. $- Estimated number of hydrogen bonds that would be accepted by the solute from water molecules in an aqueous solution.

**Table 6:** Cytotoxicity prediction using QNA, MNA and Combinatorial model.

Pubchem id	IC50	pIC50 (QNA)	pIC50 (MNA)	pIC50 (Combinatorial)
477	0.087	0.44	-3.35	-0.15
890	4.39	5.97	-0.71	3.81
107758	0.002	0.46	-3.38	-0.14
125004	20	17.68	18.64	15.38
439456	3.55	4.17	-0.23	2.85
443266	0.43	0.39	-3.03	0.04
10251645	2	0.95	-7.18	-0.01
14375662	0.268	0.95	-7.18	-0.01
16752673	19	17.89	18.12	16.67
44274820	0.009	-2.75	-27.9	-2.08
44332437	0.28	4.17	-0.23	2.85
46905360	0.172	-0.18	-4.32	-0.86

IC50- experimental IC50, p IC50- predicted IC50 N= 12, R2= 0.976, F=27.059, SD= 1.600, Q2= 0.453, V= 3 N is total number of molecules used, R is correlation coefficient, F is value of Fischer’s parameter, SD is standard deviation, the cross-validated R2 and V is no. of variables used in the model building.
